# Alternative Crassulacean Acid Metabolism Modes Provide Environment-Specific Water-Saving Benefits in a Leaf Metabolic Model

**DOI:** 10.1105/tpc.20.00132

**Published:** 2020-10-22

**Authors:** Nadine Töpfer, Thomas Braam, Sanu Shameer, R. George Ratcliffe, Lee J. Sweetlove

**Affiliations:** a Leibniz Institute of Plant Genetics and Crop Plant Research, 06466 Gatersleben, Germany; b Department of Plant Sciences, University of Oxford, Oxford OX1 3RB, United Kingdom; c Innova Solutions, Taipei City 11087, Taiwan

## Abstract

Crassulacean acid metabolism (CAM) evolved in arid environments as a water-saving alternative to C_3_ photosynthesis. There is great interest in engineering more drought-resistant crops by introducing CAM into C_3_ plants. However, it is unknown whether full CAM or alternative water-saving modes would be more productive in the environments typically experienced by C_3_ crops. To study the effect of temperature and relative humidity on plant metabolism in the context of water saving, we coupled a time-resolved diel (based on a 24-h day-night cycle) model of leaf metabolism to an environment-dependent gas-exchange model. This combined model allowed us to study the emergence of CAM as a trade-off between leaf productivity and water saving. We show that vacuolar storage capacity in the leaf is a major determinant of the extent of CAM. Moreover, our model identified an alternative CAM cycle involving mitochondrial isocitrate dehydrogenase as a potential contributor to initial carbon fixation at night. Simulations across a range of environmental conditions show that the water-saving potential of CAM strongly depends on the daytime weather conditions and that the additional water-saving effect of carbon fixation by isocitrate dehydrogenase can reach 11% total water saving for the conditions tested.

## Abstract (precis)

Stoichiometric modeling of leaf metabolism reveals metabolic and morphological determinants for introducing Crassulacean acid metabolism and alternative water-saving flux modes into a C_3_ leaf in different environments.

## INTRODUCTION

Increasing aridity threatens agricultural productivity not only in hot and dry climates but also in temperate regions where extreme weather conditions are becoming more frequent (Olesen and Bindi, 2002; Gornall et al., 2010). Thus, the development of crop varieties that use water efficiently is of utmost importance to maintain food security (Borland et al., 2014). Several plant lineages living in arid environments have evolved Crassulacean acid metabolism (CAM) photosynthesis, a water-saving mode of carbon fixation in which CO_2_ uptake into the mesophyll cell and CO_2_ fixation by ribulose-1,5-bisphosphate carboxylase/oxygenase (Rubisco) are temporally separated (Osmond, 1978). In plants performing CAM photosynthesis, the stomata open at night and CO_2_ is fixed and stored in the vacuole in the form of a carboxylic acid such as malate, citrate, or isocitrate (Maclennan et al., 1963; Lüttge, 1990; Gawronska and Niewiadomska, 2015; Igamberdiev and Eprintsev, 2016). During the hot, dry daytime hours, the stomata can remain closed to minimize water loss, and the stored CO_2_ is remobilized for fixation by Rubisco in the chloroplast, accompanied by the accumulation of storage carbohydrates. Although this cycle is energetically expensive, it conserves precious water and is an efficient alternative to direct daytime CO_2_ fixation by Rubisco as in C_3_ photosynthesis (Dodd et al., 2002; Garcia et al., 2014).

**Figure:**
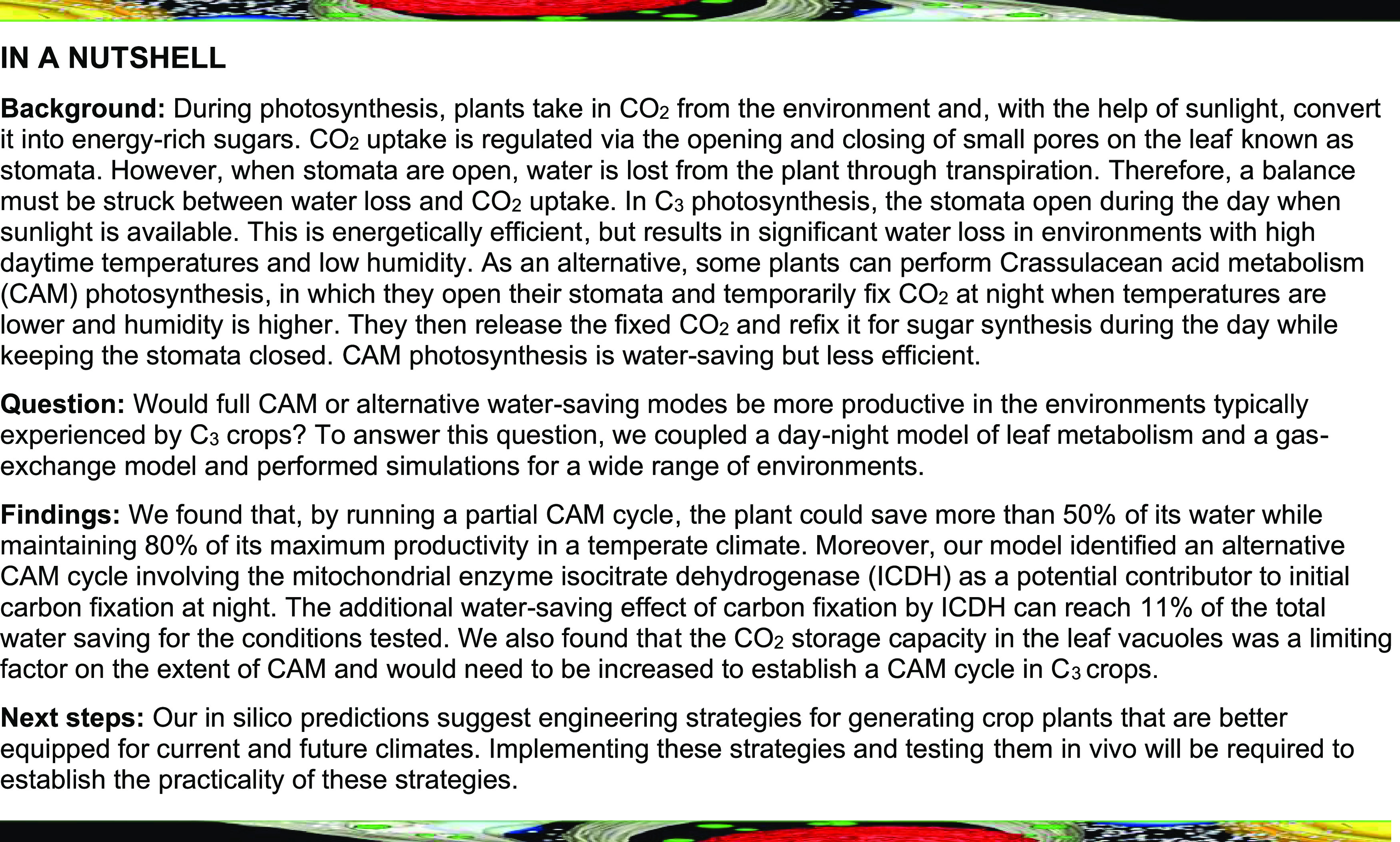


The implementation of CAM photosynthesis into a C_3_ crop plant is a promising engineering target for two reasons. First, all enzymes required for the CAM cycle are already present in C_3_ plants, although specific isoforms with different regulatory properties are required (Cushman and Bohnert, 1997). Second, some facultative CAM species, such as the ice plant (*Mesembryanthemum crystallinum*), can be induced to switch from C_3_ to CAM photosynthesis by a number of environmental factors such as drought or high salinity (Winter et al., 1978; Garcia et al., 2014). This suggests that it should be possible to engineer CAM into a C_3_ leaf. CAM photosynthesis is usually considered to be advantageous in hot and arid climates where water-use efficiency (WUE) is a strong determinant for plant growth and where the suppression of photorespiration through carbon concentration behind closed stomata becomes a considerable factor that balances the additional cost of running the energy-intensive CAM cycle (Cushman, 2001). In a previous study, we tested this hypothesis by investigating the energetics and productivity of CAM by employing a day-night flux balance analysis model simulating either CAM or C_3_ leaf metabolism. To study the effect of suppressed photorespiration behind closed stomata, we modeled CAM leaf productivity at different Rubisco carboxylation-to-oxygenation ratios and compared this with the productivity of C_3_ leaf metabolism. We found that despite threefold higher energy consumption at night, the additional cost of running a CAM cycle can be balanced by the carbon-concentrating effect of carboxylic acid decarboxylation behind closed stomata during the day (Shameer et al., 2018). However, in that work, the simple two-phase day-night model did not allow anything other than a full CAM cycle to be considered and did not take into account the range of environments in which C_3_ crops are cultivated.

In this work, we tested whether full CAM is necessarily the best solution for C_3_ crops grown in temperate environments and attempted to identify alternative metabolic modes that best balance the trade-off between water loss and photosynthetic productivity under a range of environments. To this end, we constructed a time-resolved, large-scale metabolic leaf model and coupled it to a gas-exchange model that includes the two main determinants of water loss through the stomata: the temperature (T) and the relative humidity (RH). This environment-coupled model was used to investigate emergent metabolic flux modes when water-saving constraints are imposed while still requiring the leaf to be highly productive. We found that the leaf’s vacuolar storage capacity is a major determinant of the extent of CAM and that without engineering a higher vacuole-to-cytoplasm ratio it will be unlikely to succeed in introducing a full CAM cycle into a C_3_ leaf. Moreover, mitochondrial isocitrate dehydrogenase (ICDH) might contribute to initial carbon fixation at night. Finally, simulations across a range of environmental conditions showed that the additional water-saving effect of carbon fixation by ICDH can reach 11% of the total water saving for the conditions tested.

## RESULTS

### Model Construction

Light availability and gas exchange (CO_2_ and water vapor) are major determinants of the metabolic behavior of a plant leaf. To model the interplay between leaf productivity and transpirational water loss, we extended a previous diel flux balance modeling framework (Shameer et al., 2018) in two ways. First, we increased the temporal resolution from a binary day-night scenario to modeling a 24-time-step diel cycle, in which each interval represents 1 h of the day. The time resolution in the models was achieved by coupling 24 copies of the model in series, each with a predefined list of metabolites (starch, sugars, amino acids, carboxylic acids, and nitrate; Supplemental Methods) that were allowed to accumulate in one model and then to be passed to the next in the time series. For each of these metabolites, we introduced linker reactions that transfer the accumulated metabolite from one time interval to the next. Upper bounds were placed on the quantity of carboxylic acids and other compounds that were allowed to accumulate in the vacuole based on vacuole size and leaf anatomy (leaf thickness and porosity) for average C_3_ and CAM leaves.

**Figure 1.**
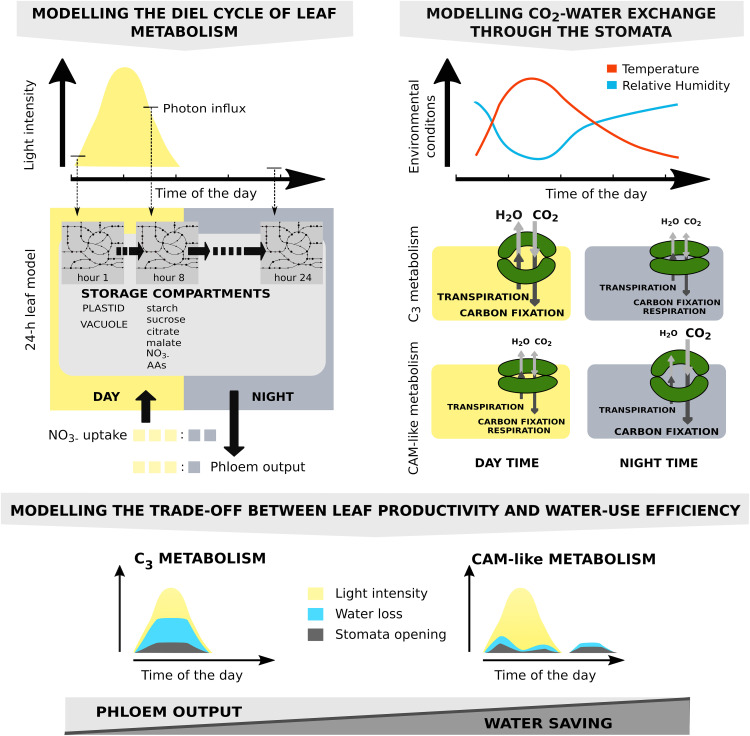
Modeling Water-Saving Flux Modes in an Environment-Coupled Model of Leaf Metabolism. **(Top left)** A diel (24-h) leaf model was constructed by concatenating copies of a core model of plant metabolism (Shameer et al., 2018). The individual models were connected via linker reactions that allowed the transfer of storage compounds in the vacuole and the plastid between successive models. Light uptake was constrained by the diel light curve. The day:night ratios of phloem output and maintenance were set to 3:1 for each hour of the diel cycle, and N uptake was constrained to a ratio of 3:2 based on previous estimates (Cheung et al., 2014). **(Top right)** The effect of T and RH on stomatal water loss was modeled by a simplified gas-diffusion equation. T and RH data determined the relationship between CO_2_ uptake and water loss. The four stomata pores illustrate the water-saving mechanism of nocturnal CO_2_ uptake. While respiration occurs in all four scenarios, dominant carbon fixation leads to a net uptake of CO_2_ during the day in C_3_ plants and at night in CAM plants. **(Bottom)** Combining metabolic and gas-exchange models allowed us to study the trade-off between productivity and water loss as competing objectives on a Pareto frontier (i.e., the line that denotes combinations of productivity and water-loss values where one objective cannot be improved without compromising the other) and revealed alternative water-saving carbon-fixation mechanisms.

The second extension to our previous diel metabolic modeling framework was the introduction of a simplified gas-diffusion model. This allowed us to compute water loss through the stomata according to the CO_2_ demand of the metabolic system and the environmental conditions (T and RH). Note that although this indirectly accounts for stomatal aperture dynamics—a decreased CO_2_ demand requires a smaller stomatal aperture and hence there is less water loss—regulatory aspects of the stomatal aperture are not included. Hence, altered diel CO_2_ uptake patterns are an emergent property of the optimization criteria of the model. A detailed description of the model construction and the exchange constraints is given in Methods. The resulting time-resolved, environment-coupled model enabled us to simulate the effect of the diel changes in light, T, and RH on leaf metabolism and was used to study the trade-offs between leaf productivity and WUE (Figure 1).

In this study, we considered the metabolism of a mature source leaf under field conditions (i.e., a metabolic system that functions to assimilate C and N and to synthesize sugars and amino acids at a defined composition for export to the phloem [phloem output] while at the same time meeting costs for cell maintenance). We assumed that mineral nutrients were not limiting, as is likely in a fertilized high-intensity agricultural system. Therefore, we started the simulations by using maximization of phloem output over the course of the day as the primary objective. This optimality criterion led the metabolic system to synthesize storage compounds in the light that were then used to sustain nighttime metabolic processes such as phloem output, maintenance, and nitrogen assimilation in an overall optimal manner. In accordance with the metabolic mode of the system, the model predicted changing CO_2_ demand and, depending on T and RH, water loss by transpiration over the course of the day (see section Model Analysis and Biological Implications below). Subsequently, we could also fix the minimum required phloem output to a given value (thereby also reducing the system’s demand for CO_2_) and could use minimization of water loss as an optimality criterion to act on the metabolic system. These constraints led to the prediction of water-saving flux modes while maintaining high productivity.

### Model Analysis and Biological Implications: A Time-Resolved Diel Model Simulates the Dynamics of C_3_ Metabolism

**Figure 2.**
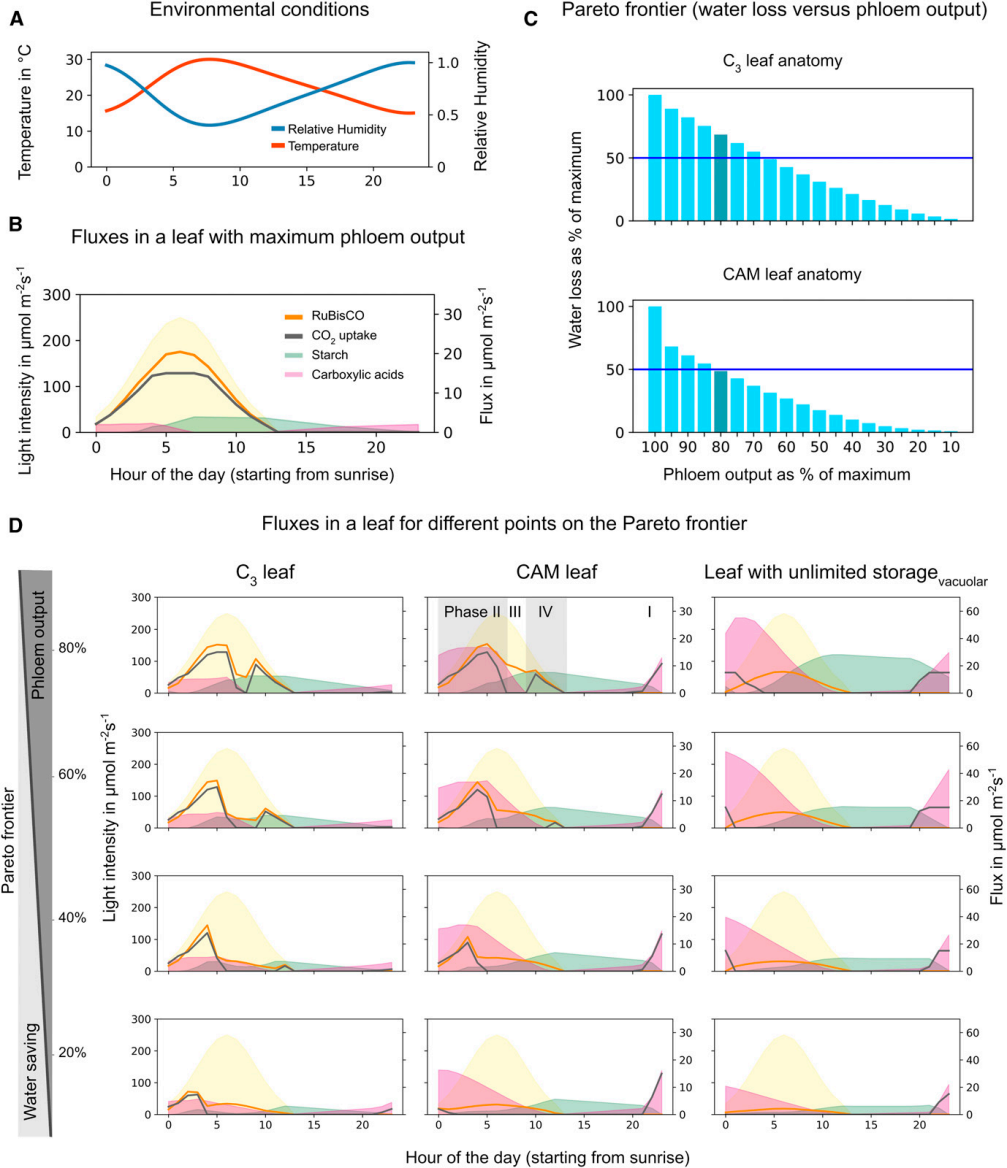
Metabolic Fluxes and Water Loss for Different Modeling Scenarios. **(A)** Example of the T and RH data used throughout the simulations. **(B)** Metabolic flux profiles in a C_3_ leaf optimized toward phloem output (100% phloem output). The diel light curve is indicated in yellow and peaks at a maximum intensity of 250 μmol m^−2^ s^−1^. **(C)** Pareto analysis of phloem output versus water loss in a C_3_ leaf (top) and a CAM leaf (bottom). The CAM leaf enabled a better trade-off between the two competing objectives. **(D)** Metabolic flux profiles for a C_3_ leaf (left), a CAM leaf (middle), and a leaf with unlimited vacuolar storage capacity for different Pareto steps (right; 80, 60, 40, and 20% of the maximum phloem output [shown for a C_3_ leaf in **(B)**]). Note the different flux scales on the right plot axis for the C_3_ and CAM leaves and the leaf with unlimited storage capacity.

To establish a reference model for a mature source leaf operating under optimal conditions, we began our analysis without imposing water-saving constraints. We simulated a typical summer day in a temperate climate by using T and RH curves with a maximum T of 30°C and a minimum RH of 0.4, values that were based on measurements from the IPK weather station in Gatersleben, Germany (Supplemental Figure 1; Supplemental Methods, Section 1.2.2). To simulate light intensities inside a canopy, we used a normally distributed light curve that peaks at midday at a moderate intensity of 250 μmol m^−2^ s^−1^. The daylength was set to 12 h (Figure 2A). The primary optimization objective was maximization of phloem output; a second optimization criterion was subsequently applied to minimize the metabolic flux sum (Holzhütter, 2004). This objective was used as a proxy for the cost of providing the enzymes for the active reactions. Applying it as a second optimality criterion did not alter the primary objective value, the phloem output, but chose the flux distribution with the lowest sum of fluxes from a set of alternatives.

Using this setup, the model predicted a phloem output of 1.0 mol m^−2^ leaf d^−1^. Daily total water loss was predicted to be 2.6 × 10^3^ mol m^−2^ leaf and CO_2_ uptake was 10.7 mol m^−2^ leaf. This resulted in 249.8 mol of water lost per mol of CO_2_ fixed. For more extreme conditions with a light intensity of 1200 μmol m^−2^ s^−1^, a maximum T of 40°C, and a minimum RH of 0.1, this value increased to 753.8 mol of water lost per mol of CO_2_ fixed (Supplemental Data Set; Supplemental Results, Section 2.3, presents a sensitivity analysis of water loss to varying internal CO_2_ concentrations). Comparing this value with an average value for C_3_ plants, 900 to 1200 mol of water lost per mol of CO_2_ fixed (Smil, 2003), we found that our model is in broad agreement with experimental observations. This was reassuring, given the simple nature of the gas-exchange model and the fact that water loss was predicted from the model’s demand for CO_2_ to maximize phloem output, thereby justifying the choice of the objective function.

To get a better overview of the metabolic behavior over the course of the diel cycle, we examined the CO_2_ uptake, Rubisco activity, and linker fluxes for starch and carboxylic acids (Figure 2B). The magnitude of a linker flux corresponds to the amount of the stored metabolite: a flux of 1 μmol m^−2^ s^−1^ means that 3.6 mmol m^−2^ is available (given that each time interval is 1 h) for utilization in subsequent time intervals in the model. Both CO_2_ uptake (gray line) and Rubisco flux (orange line) were predicted to follow the light curve and peaked at midday, coinciding with light availability. Carboxylic acid levels (magenta area) peaked before midday, decreased until early afternoon, and slowly increased from before sunset to dawn. Starch (green area) accumulated during daytime hours and was subsequently degraded to sustain metabolism at night. Overall, the predicted flux patterns are characteristic of C_3_ leaf metabolism. From this starting point, we then asked the question: How will the metabolic fluxes change if we alter the optimality criterion from maximizing phloem output to that of minimizing water loss?

### An Optimality Study Reveals Trade-Offs between Productivity and WUE

Computationally, the question of how the behavior of a system changes when operating between competing objectives can be tackled by performing a Pareto analysis: a decision-making technique to identify trade-offs and the impact of increasing one objective’s value on the other (Laundy and Steuer, 1988; Farnsworth and Niklas, 1995; Kennedy, 2010; Cheung et al., 2013). In our case, phloem output and water saving represented two competing driving forces. We started the Pareto analysis from the scenario described above: a mature leaf optimized for phloem output (i.e., 100% phloem output, hereby termed Pareto step 1). We then subsequently reduced the required phloem output in 5% steps and used minimization of water loss as the primary optimization objective. Given this setup, we saw an almost linear decrease in water loss with decreasing phloem output, and thus no significant water-saving mechanism that would increase the ratio of phloem output to water loss (Figure 2C, top). Inspection of the CO_2_ uptake and Rubisco reaction flux in the model showed that with decreasing phloem output, the model predicted a cessation of CO_2_ uptake (suggesting a closure of stomata) during the warmest and driest hours of the day, a phenomenon known as midday depression of photosynthesis (Figure 2D, left column), which was accompanied by a minor peak of CO_2_ uptake toward the end of the night.

One possible explanation for the lack of water-saving metabolic modes in the model was that the model was limited by the constraints we applied to mimic C_3_ leaf anatomy (e.g., total vacuolar volume per unit of leaf). To test this, we examined the differences between C_3_ and CAM leaf anatomy and adjusted the vacuolar storage constraints accordingly. Using morphological data for an average CAM leaf resulted in a 3.1-fold increase in vacuolar storage capacity per unit of leaf compared with a C_3_ leaf (Supplemental Methods, Section 1.2.4). When repeating the Pareto analysis using this CAM morphology, a nonlinear relationship between productivity and water loss emerged and the model predicted more than 50% water saving at 80% of the maximum phloem output. This was an increase of 19.8% in water saving with respect to C_3_ morphology (Figure 2C). It is worth noting that the upper limit for the vacuolar storage capacity had only a very minor impact on the maximum phloem output of the model. The output at Pareto step 1 for the C_3_ leaf model was 99.6% of the phloem output of the CAM leaf. Therefore, in subsequent analyses, we directly compared between the two sets of simulations.

What was causing the nonlinearity in the relationship between productivity and water loss? As in the C_3_ anatomy-constrained model, we observed reduced CO_2_ uptake and Rubisco activity during the hottest and driest hours of the day. However, in addition to these daytime changes to minimize water loss, we also observed a substantial peak of CO_2_ uptake at the end of the night that was accompanied by an accumulation of carboxylic acids in the vacuole and a greater amount of starch stored during the day and degraded at night (Figure 2D, middle column). These observations suggested that the model was performing a CAM or CAM-like cycle in which CO_2_ was initially fixed at night and stored in the form of carboxylic acids. During the day, when sufficient light energy was available, CO_2_ was released from its intermediate storage and refixed for triose phosphate synthesis during the day using Rubisco and the Calvin-Benson-Bassham (CBB) cycle. This was confirmed by inspection of the complete set of predicted fluxes in the model (Supplemental Results, Section 2.1). Note that the results described thus far are not sensitive to the time interval of the model: quantitatively similar results were obtained when the model was run again with 2-, 4-, or 6-h time intervals (Supplemental Results, Section 2.5).

When inspecting the Pareto frontier, we observed the steepest slope (i.e., the largest increase in water saving) between 95 and 100% of the maximum phloem output. At 95% maximum phloem output, the model had already switched to CAM-like behavior and predicted a partial cessation of CO_2_ uptake during the day and resumption for a short period at night. Comparing this with the almost linear Pareto frontier for the C_3_ leaf indicated that the additional effect of nighttime CO_2_ fixation contributed largely to the water saving.

### Vacuolar Storage Capacity Limits WUE and Influences the Extent of Phases II and IV of the CAM Cycle

When investigating the CO_2_ uptake at different steps along the Pareto frontier, it became apparent that the model did not exhibit a full CAM cycle (Figure 2D, middle column). CO_2_ uptake ceased for only a few hours in the day, and it resumed only for a short period toward the end of the night. The CO_2_ uptake at nighttime coincided with the maximum RH and minimum T, which were reached just before sunrise. This can be explained by the anticipatory nature of our model, where the solution for time point *t* depends on the environmental parameters to be encountered at time point *t* + 1. In a real CAM plant, we would expect nighttime CO_2_ uptake to be more distributed across the cooler and more humid nighttime hours.

During the day, CO_2_ uptake continued during the early hours of the day and resumed in the evening hours before sunset. This behavior was exhibited for all Pareto steps with more than 30% of the maximum phloem output, meaning that nighttime CO_2_ fixation alone was not sufficient to sustain the required phloem output. The observed CO_2_ uptake pattern, which is consistent with opening and reopening of the stomata, during the day occurs in certain CAM species and is known as phases II and IV of the CAM cycle (Osmond, 1978). Nighttime stomata opening for CO_2_ uptake and daytime stomata closure are referred to as phases I and III (Table 1), respectively. Some CAM species show a remarkable plasticity with respect to these four phases, and the reasons for the occurrence and extent of these distinct patterns are still debated (Cockburn, 1985; de Mattos et al., 1999; Lüttge, 2004). Given the indication that vacuolar storage capacity had a major impact on the nighttime CO_2_ uptake pattern in the model and the fact that some CAM species exhibit a biphasic CAM cycle, we wondered whether we might have underestimated the vacuolar storage capacity of an average CAM leaf. We therefore repeated the Pareto analysis using the same model but without any vacuolar storage constraints. The results of this analysis are shown in Figure 2D, right column. In the absence of any limitation of the vacuolar storage capacity, the model performed a biphasic full CAM cycle (i.e., the CO_2_ uptake was limited to the night) without the appearance of phase II or IV of the CAM cycle. Therefore, the model suggested that continued CO_2_ uptake for at least a portion of the day was necessary to sustain a high productivity when vacuolar storage capacity is limiting.

**Table 1.** Four Phases of the CAM Cycle

Phase	Description
I	Stomata open during the dark period and nocturnal CO_2_ assimilation by PEPC
II	Transition phase between dark and light periods with a peak in CO_2_ uptake and fixation of CO_2_ by Rubisco
III	Stomata closed during the light period and fixation of CO_2_ that is released by decarboxylation of carboxylic acids
IV	Transition phase between light and dark periods with direct Rubisco-mediated fixation of CO_2_

### Nighttime Carbon Fixation by ICDH Contributes to WUE

**Figure 3.**
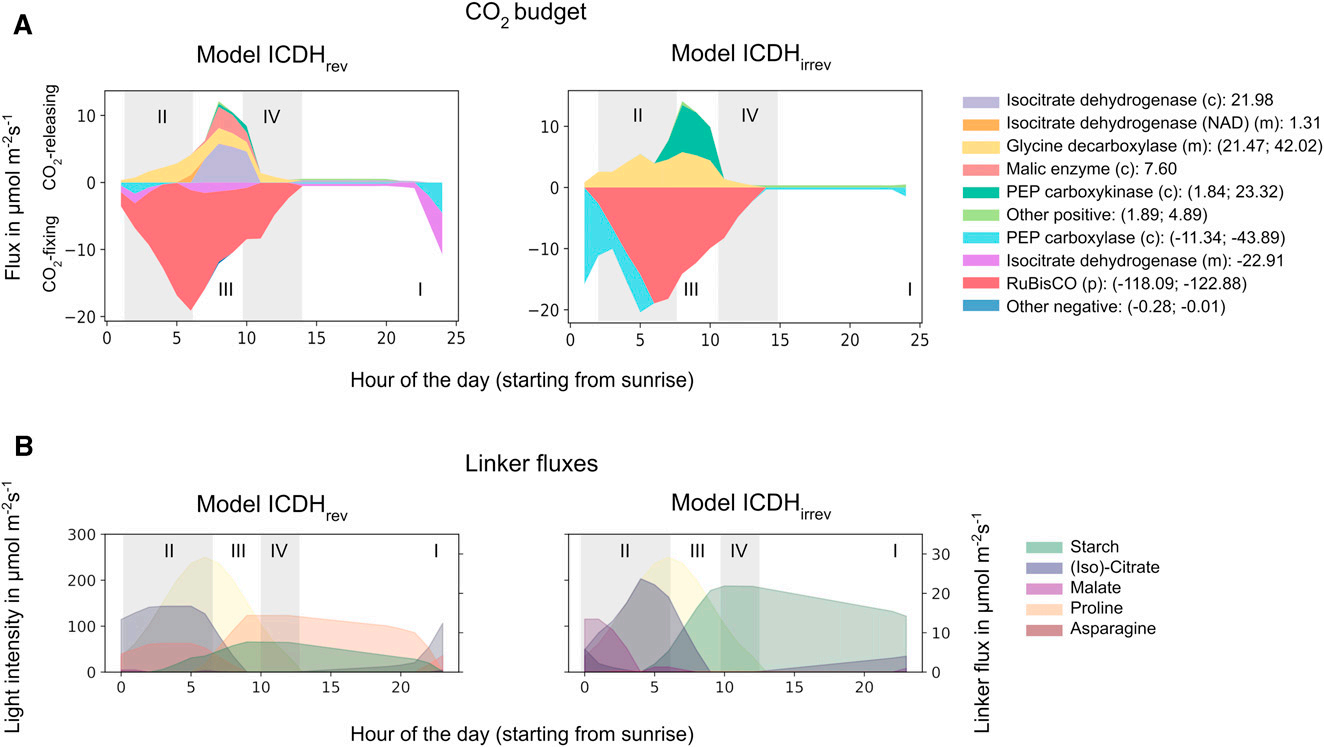
Different Flux Distributions in a Water-Saving CAM Leaf at 80% Productivity with (Model ICDH_rev_) and without (Model ICDH_irrev_) Reversible Mitochondrial ICDH. **(A)** CO_2_ budget for the two models reveals different CO_2_ turnover fluxes over the course of the day. Shown are all reactions with flux > 0.5 μmol m^−2^ s^−1^ for at least one time point. I to IV indicate the four phases of the CAM cycle. The values for the cumulative contribution are given next to the reaction name for either model ICDH_rev_ or both models (model ICDH_rev_ and model ICDH_irrev_). c, cytosolic; m, mitochondrial; p, plastidial. **(B)** Significant linker fluxes for both models. Model ICDH_rev_ accumulated (iso)citrate as carboxylic acid and additionally Pro and Asp. Model ICDH_irrev_ accumulated both malate and (iso)citrate but no amino acids. Starch levels in model ICDH_irrev_ were almost threefold higher than in model ICDH_rev_.

The occurrence of the four phases of CAM in the model raised the following question: How were metabolic fluxes distributed during these metabolically distinct phases? To analyze the underlying flux modes in more detail, we focused the analysis on a model with the vacuolar storage capacity of a CAM leaf at 80% of maximum productivity (phloem output) optimized for water saving. We chose this value because a yield penalty of 20% would be an acceptable trade-off if water usage could be reduced by more than half. We followed the flux of CO_2_ (including bicarbonate) from the stomata through the metabolic system by plotting time-resolved fluxes of all reactions that use CO_2_ or bicarbonate as either a reactant or a product (Figure 3A, left). During the day, Rubisco fixed the majority of CO_2_ available from gas exchange and released by metabolic processes. Cytosolic ICDH, Gly oxidation in the photorespiratory pathway (Gly decarboxylase), and NADP-malic enzyme in the cytosol were the main CO_2_-releasing processes during the day.

To our surprise, we found that nighttime CO_2_ fixation in the model was shared between two enzymes—phospho*enol*pyruvate carboxylase (PEPC) in the cytosol and ICDH in the mitochondria. While the role of PEPC in CAM photosynthesis is well established, mitochondrial ICDH activity has not been previously linked to this metabolic cycle. In order for ICDH to be used for CO_2_ fixation, it has to operate in the reverse of its conventional direction in the tricarboxylic acid (TCA) cycle. This is possible given an appropriate mass action ratio (e.g., due to a high 2-oxoglutarate [2OG] concentration), and indeed this reaction has been shown to operate in the reverse direction in several in vivo metabolic flux studies in developing rapeseed (*Brassica napus*) and soybean (*Glycine max*) embryos (Schwender et al., 2006; Allen et al., 2009; Allen and Young, 2013). ICDH has also been suggested as a kinetically acceptable option for synthetic carbon fixation pathways (ΔG = 21 kJ mol^−1^ at pH 7, ionic strength of 0.1 M, and reactant concentrations of 1 mM; Bar-Even et al., 2010, 2012). For convenience, we refer to this reaction as ICDH_rev_.

Analysis of the linker fluxes revealed that citrate and/or isocitrate were the main carboxylic acids accumulating at night. Accumulation of either citrate or isocitrate or of both carboxylic acids resulted in the same phloem output and water saving. Additionally, two amino acids accumulated to high levels: Asn during the night and Pro during the day (Figure 3B, left). Additionally, Glu accumulated at lower levels at night. None of the other linker reactions in the vacuole carried a significant flux.

**Figure 4.**
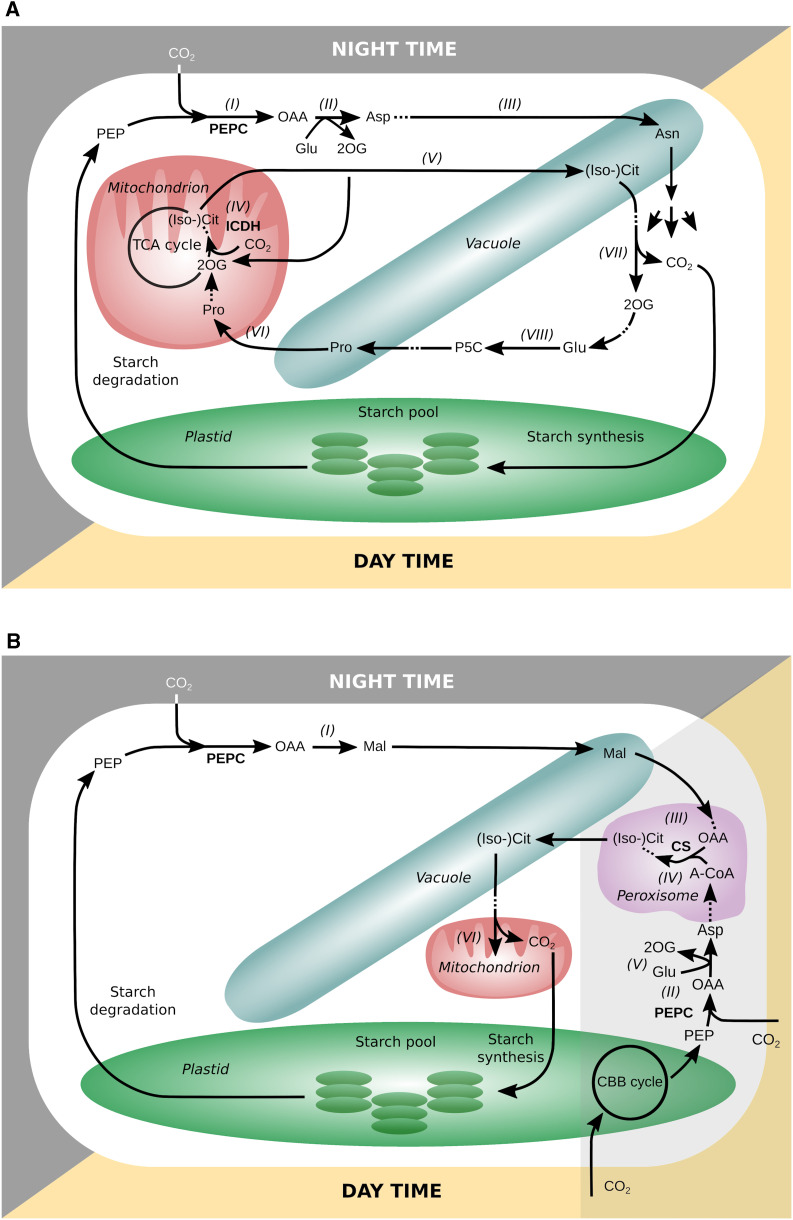
Major Flux Routes Involved in the CAM-Like Temporally Separated Carbon-Fixation Mechanism in a Water-Saving CAM Leaf at 80% Productivity. Analysis with reversible mitochondrial ICDH (model ICDH_rev_; **[A]**) and analysis without reversible mitochondrial ICDH (model ICDH_irrev_; **[B]**) are shown. The two models used different pathways to fix and release CO_2_. A-CoA, acetyl-CoA; CS, citrate synthase; (Iso-)Cit, (iso)citrate; Mal, malate; P5C, 1-pyrroline-5-carboxylic acid. The gray area in **(B)** highlights those reactions that are active in phase II. Roman numerals indicate the sequences of reactions described in the text.

Closer inspection of the metabolic fluxes revealed an alternative CO_2_ fixation pathway in which both PEPC and ICDH contributed to nighttime CO_2_ fixation. An overview of the reactions involved is shown in Figure 4A. At night, PEPC catalyzed the fixation of CO_2_ to PEP [marked as (I) in Figure 4A]. The resulting oxaloacetate (OAA) together with Glu was converted to 2OG and Asp by Asp aminotransferase (II). Asp was converted to Asn and stored in the vacuole (III). 2OG was translocated to the mitochondria as a substrate for ICDH_rev_ to catalyze the carboxylation of 2OG to isocitrate (IV), which was either directly stored in the vacuole or further converted to citrate and then stored in the vacuole for daytime usage (V). Additionally, conversion of the vacuolar pool of Pro to 2OG supported the flux through ICDH in the mitochondria (VI). In total, this pathway—from PEP to the carboxylic acids stored in the vacuole—can fix 1 mol of CO_2_ per mol of stored (iso)citrate or Asn. During the daytime, the (iso)citrate from the vacuole was converted to 2OG and CO_2_ by cytosolic ICDH, and CO_2_ was refixed in the CBB cycle (VII) and ultimately stored as starch to then support night-time metabolism. 2OG was converted to Pro and stored in the vacuole for the next nighttime period (VIII).

This flux mode raised the following questions: Does the shared nighttime carbon fixation between PEPC and ICDH represent an advantage? And, if so, to what extent is it more beneficial than using PEPC alone? To answer these questions, we set ICDH to be irreversible in the conventional forward direction (ICDH_irrev_) and ran the simulations again. We found that the carboxylating activity of ICDH increased water saving by 1.7% (for this particular set of parameters). From these observations, we concluded that nighttime carbon fixation by ICDH_rev_ in combination with daytime storage of Pro as a precursor for 2OG might act as an additional water-saving mechanism by adding to the temporal separation of initial CO_2_ fixation and the activity of the CBB cycle.

### PEPC Also Fixes CO_2_ in the Early Hours of the Day When ICDH Is Irreversible

How do the metabolic fluxes in our model differ when ICDH_rev_ is not available for carbon fixation, and how does it affect WUE? A first inspection of the metabolic fluxes for this scenario revealed that making ICDH irreversible had a major impact on the accumulation pattern of both carboxylic acids and starch (Figure 3B, right). While the overall diel pattern of carboxylic acid accumulation and degradation was similar in the two scenarios, the individual patterns for malate and (iso)citrate were different. When ICDH was irreversible, we observed low-level (iso)citrate accumulation at night and a sharp increase of malate around sunrise that was followed by a drop in the early morning together with a strong increase of (iso)citrate. Daytime starch levels were more than twice as high as in the scenario where ICDH was reversible, and the onset of starch accumulation was shifted toward the later hours of the morning. Pro and Asn did not accumulate. Closer inspection of the flux routes involved in the carbon-fixation cycle revealed differences between the two scenarios (Figure 4B). Nighttime CO_2_ uptake only started at the end of the night. The (iso)citrate levels increased during the night due to the use of internally released CO_2_. At the end of the night and the onset of sunrise, PEPC fixed CO_2_ to PEP and formed OAA, which was mainly converted to malate and stored in the vacuole (I). Net, this pathway, from PEP to the carboxylic acid stored in the vacuole, can fix 1 mol of CO_2_ per mol of stored malate.

During the day, the two models showed marked differences in the flux routes between phase II and phase III of the CAM cycle. While model ICDH_rev_ predicted that CO_2_ fixation through PEPC was mainly limited to the nighttime, model ICDH_irrev_ showed additional PEPC activity during phase II in parallel with Rubisco activity (Figure 3A, right). This early-morning PEPC activity increased the amount of CO_2_ that could be transiently stored in the vacuole until sufficient light energy was available for starch synthesis. PEPC used PEP delivered from the CBB cycle as a substrate (II) to generate OAA. At the same time, malate was released from the vacuole and converted to OAA by malate dehydrogenase in the peroxisome (III). A part of the OAA pool and acetyl-CoA were substrates for citrate synthase in the peroxisome (IV). The other part of the OAA pool, together with Glu, was used by an aminotransferase to generate 2OG and Asp in the cytosol (V). Asp was further metabolized, and the downstream product acetyl-CoA (see sequence of reactions in Supplemental Results, Section 2.1) acted as a precursor for the synthesis of citrate by citrate synthase and (iso)citrate replaced malate in the vacuole. PEPC is known to be active in phase II (Borland et al., 1993; Roberts et al., 1997); however, subsequent metabolic flux modes in the model were different from the canonical CAM cycle in which malate is decarboxylated to PEP or pyruvate by PEP carboxykinase or malic enzyme. Later during phase III, (iso)citrate was released from the vacuole and supplied CO_2_ for the CBB cycle via a degradation route that involved the Gly decarboxylase system in the mitochondria (VI; see reaction sequence in Supplemental Results, Section 2.1). To test whether the observed malate-to-(iso)citrate exchange in the vacuole during phase II of the CAM cycle was indeed a water-saving advantage, we simulated a scenario in which (iso)citrate uptake into the vacuole was blocked during the day (termed model ICDH_irrev, Cit_night_). This constraint increased water usage at 80% productivity but by only 0.5%.

### High Enzyme Costs Might Outweigh the Water-Saving Effect of Alternative Flux Routes

The occurrence of specific metabolic patterns was determined not only by WUE but also by the cost for enzyme synthesis. An accurate description of these processes at a large scale is currently limited to microbial systems for which sufficient data are available and for which enzyme turnover rates can be neglected due to high doubling times (Goelzer et al., 2015; Lantier et al., 2015; Rügen et al., 2015; Reimers et al., 2017; Bulović et al., 2019). An established alternative is the minimization of the sum of metabolic fluxes (Holzhütter, 2004; Lewis et al., 2010). In our analysis, the enzyme investment was considered by minimizing the metabolic flux sum after the leaf productivity and water loss had been determined. Therefore, flux minimization did not represent a competing objective on the Pareto frontier, and a slightly more water-efficient solution with a high enzymatic investment (high flux sum) would always be preferred over a slightly worse-performing mechanism with less enzyme investment. To account for this bias, we considered the metabolic flux sum for the three models: ICDH_rev_, ICDH_irrev_, and ICDH_irrev, Cit_night_. The values were 9098, 9670, and 9126 μmol m^−2^ s^−1^, respectively.

As a second indicator for metabolic efficiency, we considered the overall ATP budget (i.e., all ATP produced and consumed over the course of the day; Supplemental Figure 2). These values indicated the highest ATP turnover of 869 μmol m^−2^ s^−1^ for model ICDH_irrev_ and lower values of 807 and 811 μmol m^−2^ s^−1^ for models ICDH_rev_ and ICDH_irrev, Cit_night_, respectively. From these observations, we conclude that the additional metabolic cost of exchanging malate for (iso)citrate in phase II of the CAM cycle would very likely outweigh the water-saving effect of this mechanism. On the other hand, the other two modeling scenarios, ICDH_rev_ and ICDH_irrev, Cit_night_, had similar metabolic flux sums as well as ATP budgets, indicating that the contribution of ICDH to CO_2_ fixation is a feasible prediction with respect to enzyme cost.

### CAM WUE Depends on the Environment

**Figure 5.**
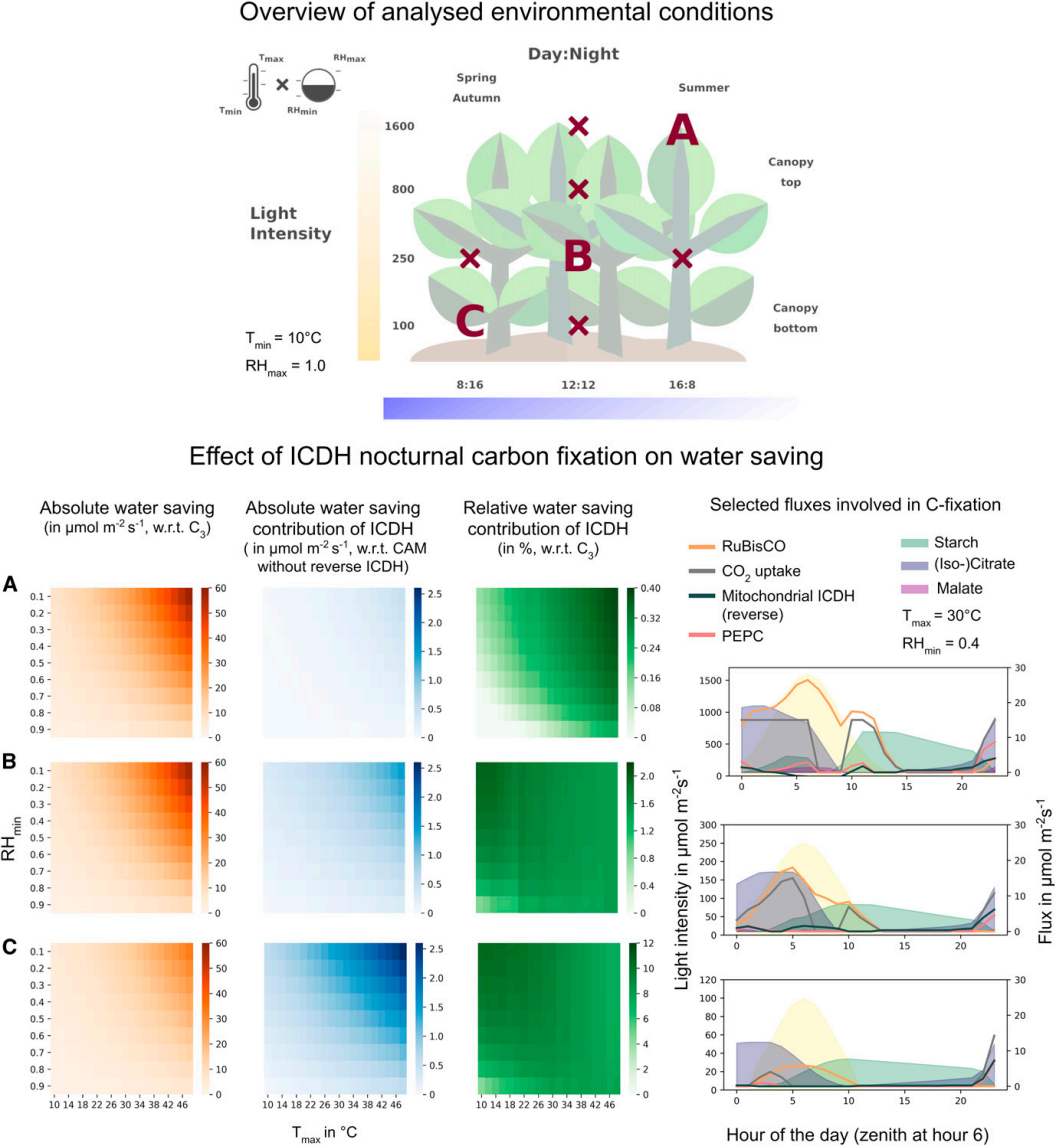
Water Saving of a Leaf with CAM-Like Nocturnal Carbon Fixation by ICDH at 80% Productivity for Different Environments. **(Top)** Overview of the environmental conditions analyzed. The T_max_-RH_min_ space was analyzed for different combinations of light intensity and daylength. Conditions A, B, and C are shown below. Conditions with an x are shown in Supplemental Results, Section 2.3. **(Bottom)** Shown are heat maps for the absolute water saving of model ICDH_rev_ with respect to the C_3_ scenario (orange), the absolute water-saving contribution of ICDH (i.e., the difference in water saving between model ICDH_rev_ and model ICDH_irrev_; blue), and the relative water-saving contribution of ICDH with respect to the C_3_ scenario (green; note the different scaling of the color bar) for combinations of T_max_ and RH_min_. Also shown are representative fluxes involved in carbon fixation and the shared nocturnal carbon fixation by PEPC and ICDH for the different environments at T_max_ = 30°C and RH_min_ = 0.4 (right column).

So far, we have focused our analysis on one particular environmental scenario. In the next step, we used the model to study the impact of different environments on the trade-off between productivity and WUE, focusing on two questions: In which environments is the introduction of a CAM cycle most beneficial? In which environments is the contribution of nighttime CO_2_ assimilation via ICDH to water saving the greatest? To systematically scan the space of possible environments, we chose four alternative light regimes representing cloudy to sunny days and/or the gradient of light intensity within a closed-canopy cropping system: low light = 100 μmol m^−2^ s^−1^; normal light = 250 μmol m^−2^ s^−1^; high light = 800 μmol m^−2^ s^−1^; and very high light = 1600 μmol m^−2^ s^−1^. We also used three different photoperiods representing the seasons from early spring/late autumn to summer: day:night (in h) = long days (16:8); medium days (12:12); and short days (8:16). For each of these scenarios, we analyzed combinations of RH_min_ and RH_max_ between 0.4 and 1.0 across a T regime between 10 and 48°C (Figure 5, top; Supplemental Data Set) for both models ICDH_irrev_ and ICDH_rev_ at 80% of the maximum phloem output. We determined water saving with respect to C_3_ metabolism (i.e., 100% productivity) and the water-saving contribution of running the isocitrate-citrate-Pro-2OG cycle with respect to the CAM cycle without carbon fixation by ICDH. An overview of all calculated parameters across all investigated conditions can be found in Supplemental Figures 3 and 4.

From these analyses, we made the following general observations. First, we found that, for the environmental conditions we investigated and using a productivity penalty of 20%, a CAM cycle with nighttime carbon fixation by ICDH can result in relative water saving between 25.4 and 92.4% with respect to a C_3_ leaf. Under certain conditions, CAM with nighttime carbon fixation by ICDH could save up to 25% more water than CAM without nighttime carbon fixation by ICDH. This difference can make up to 10.6% of the total water saving. Second, we found that T_max_ and RH_min_, which mainly determine daytime weather conditions, had much stronger effects on water saving than T_min_ and RH_max_, which mainly determine nighttime conditions. This behavior can be explained by the different CO_2_ uptake profiles of the C_3_ and CAM scenarios and the gas-diffusion relationship between the system’s demand for CO_2_ and the resulting water loss through transpiration. Whereas any closure of the stomata during the day results in high water saving, opening stomata at night causes much less water loss. Therefore, it is primarily the extent of daytime stomata closure that drives water saving. Third, both daylength and light intensity had impacts on water saving. Interestingly, while the highest absolute water saving was seen for long days and high T, the additional water-saving contribution of the isocitrate-citrate-Pro-2OG cycle was strongest for short days and lower T.

The interaction between daylength and light intensity on the contribution of nocturnal ICDH carbon fixation to water saving is illustrated for three specific growth scenarios in Figure 5, as follows: (A) a leaf on a sunny day at the top of the canopy with very high light intensities and long days (16:8); (B) a leaf on a cloudy day and/or in the middle of the canopy with normal light intensity on a 12:12 day:night cycle; and (C) a leaf on a cloudy day and/or at the bottom of the canopy with low light and short days (8:16). Figure 5, bottom, shows the absolute water saving for model ICDH_rev_ with respect to C_3_ metabolism (orange), and the absolute (blue) and relative (green) water-saving contributions (i.e., model ICDH_irrrev_ − model ICDH_rev_) of nighttime carbon fixation by ICDH for these three scenarios. Representative fluxes involved in carbon fixation for a selected T and RH combination are also shown for each of the three environments.

In scenario A under very high light and long days, we found water saving of up to 58.0 mmol m^−2^ s^−1^ (left column) or 43.3% with respect to C_3_. Under these conditions, the contribution of ICDH was 0.52 mmol m^−2^ s^−1^ (middle column) or 0.4% with respect to C_3_ (right column). In scenario B with a 12:12 day:night cycle and moderate light intensities, we found similar water saving of up to 56.8 mmol m^−2^ s^−1^, representing up to 57.9% of the C_3_ scenario. The contribution of ICDH to total water saving reached up 1.5 mmol m^−2^ s^−1^ or 2.0% with respect to C_3_. For the short-day and low-light scenario C, water saving could reach 33.3 mmol m^−2^ s^−1^ or 92.4% with respect to C_3_, with carbon fixation by ICDH contributing up to 2.6 mmol m^−2^ s^−1^ or 10.6% with respect to C_3_. Although the CAM cycle dramatically increased water-saving efficiency in scenario C, its absolute benefit was lower due to the low overall water loss. For all three conditions, the flux patterns of PEPC and ICDH were similar in shape and magnitude. This was driven primarily by the limited CO_2_ uptake rate and the limited storage capacity of the vacuole. For the same scenarios with unlimited storage capacity, we observed prolonged nocturnal activity of up to 6 h for both PEPC and ICDH.

To summarize, we found that the introduction of a CAM or CAM-like cycle could be beneficial across a large range of conditions (normal light to very high light and normal days to long days) that are typically encountered by a crop plant in temperate and hot climates. The contribution of ICDH to water saving is greatest for low-light and short-day conditions and can reach up to 10.6% of the total water saving; however, under these conditions, the absolute water-saving effect remains moderate due to overall reduced water loss.

## DISCUSSION

Our time-resolved, environment-coupled model of leaf metabolism allowed us to study the trade-offs between productivity and water saving for different network configurations and across different environmental conditions in a systematic manner. The analysis led to three main conclusions. First, the vacuolar storage capacity of the leaf is a major determinant of the extent of the CAM cycle and, without engineering a higher vacuole-to-cytoplasm ratio, it is unlikely that a full CAM cycle can be engineered into a C_3_ leaf. Second, the reversibility of mitochondrial ICDH might contribute to initial carbon fixation at nighttime. This operational mode of the TCA cycle was previously demonstrated by metabolic flux analysis in rapeseed and soybean embryos (Schwender et al., 2006; Allen et al., 2009), but it is a novel prediction with respect to nocturnal CO_2_ assimilation. Third, the water-saving effect of CAM strongly depends on the environment, and the additional water-saving effect of carbon fixation by ICDH can reach up to 25.0% and make up to 10.6% of the total water saving for the environmental conditions tested here. The additional water-saving contribution is largest at lower light intensities and for broad ranges of T and RH—conditions typically encountered by C_3_ crops in temperate climates—and this makes introducing the isocitrate-citrate-Pro-2OG cycle a promising candidate for metabolic engineering.

### Reduced Photorespiration due to Daytime Stomata Closure Can Increase the Water-Saving Potential of CAM Leaves

In previous work on CAM photosynthesis, we investigated the energetics and productivity of metabolic networks operating in C_3_ and CAM. We found that, depending on the rates of the carboxylase and oxygenase activities of Rubisco, the productivity of a CAM network could reach between 74 and 100% of the productivity of a C_3_ network (Shameer et al., 2018). In the analysis presented here, we focused on the water-saving potential of CAM without considering the potentially positive effect of carbon concentration behind closed stomata during the day. As we do not know how the carboxylation-to-oxygenation ratio changes as we move along the Pareto frontier and during the daytime, we used a constant value of 3:1 (Ma et al., 2014). Therefore, the implications of our analysis can be regarded as a conservative estimate. Due to the suppression of photorespiration in a leaf operating in CAM mode, the actual water-saving potential at the same productivity level is expected to be higher than calculated here.

### ICDH Might Play a Role in Facultative CAM Photosynthesis

Diel cycles of Pro accumulation have been previously observed in ice plants exposed to CAM-inducing salt stress. Under this stress condition, Pro is known to act as an osmoprotectant. It has been reported that Pro accumulation proceeded in an oscillating manner in which high levels of Pro accumulated during the day (up to 16 μmol g^−1^ fresh weight), followed by a partial degradation at night that led to steadily increasing Pro levels during the CAM-induction phase (Sanada et al., 1995). The increase in Pro levels was accompanied by an increase in PEPC mRNA up to 10 d after stress exposure, a time when PEPC mRNA had reached a full CAM level. This oscillatory behavior led the authors to make the following statement: “Changes of proline in light and darkness suggested that proline plays an important role in addition to serving as an osmolyte.” However, they offered no further explanation of what this role could be. We suggest that, in addition to its function as an osmoprotectant during the day, Pro degradation at night might support carbon fixation by supplying the substrate 2OG for citrate synthesis through mitochondrial ICDH. Once PEPC capacity has been induced to the level required for full CAM, the initial CO_2_ fixation proceeds via this enzyme, as it is kinetically superior, catalyzing a thermodynamically favorable reaction compared with ICDH_rev_ (ΔG = −40 kJ mol^−1^ at pH 7, ionic strength of 0.1 M, and reactant concentration of 1 mM; Bar-Even et al., 2012).

This conclusion is further supported by another study in ice plants in which malate, citrate, and isocitrate levels and CAM-relevant enzyme activities were measured for the same CAM-inducing conditions (Gawronska and Niewiadomska, 2015). The authors reported malate and citrate levels of up to 27.5 and 29.4 mM, respectively. Isocitrate levels were ∼10% of citrate levels. These observations are within the range of our model’s predictions of carboxylic acid storage (see the comment in Supplemental Methods, Section 1.2.1). The phenomenon of (iso)citrate accumulation in CAM plants is well established, and a review by Lüttge (1988) discussed its possible ecophysiological functions, its role in nocturnal CO_2_ storage, energetic considerations that favor citric acid accumulation, carbon recycling, and osmotic consequences. To elucidate the specific role of citrate in the ice plant and to shed light on the underlying metabolic flux modes during the CAM induction period in facultative CAM, additional experiments are required in order to trace the fate of labeled CO_2_, particularly its incorporation into amino and carboxylic acids, as well as the levels of those metabolites and genetic manipulation of the (iso)citrate cycle.

### Implications for Engineering CAM into a C_3_ Species in Temperate Climates

Strategies for introducing CAM into crop plants to make them more resilient to hotter and drier conditions have been largely discussed in the context of arid or marginal lands (Borland et al., 2009, 2014; Yang et al., 2015; Wai et al., 2019). Less attention has been given to the question of whether CAM could benefit the productivity of C_3_ species such as wheat (*Triticum aestivum*) or barley (*Hordeum vulgare*) that are typically grown in temperate climates, where hot and dry periods are becoming increasingly frequent (Lopez et al., 2018; Rasmijn et al., 2018). In this context, a flexible CAM (i.e., a C_3_ + CAM phenotype) could be beneficial, as it combines high productivity in the C_3_ mode with increased WUE in the CAM mode. In addition, partial or weak CAM, in which only a modest amount of nocturnal CO_2_ accumulation occurs and the stomata are open for some of the day (Edwards, 2019), could be beneficial in temperate environments.

Naturally occurring CAM has two characteristics that make it a suitable target for engineering approaches for crops grown in temperate regions. First, CAM is extremely flexible. It has been shown that the contribution of CAM to diel CO_2_ uptake patterns can range between 0 and 100%, particularly in plants with either an ontogenetically or an environmentally induced transition from C_3_ to CAM (Winter, 2019). Second, CAM has evolved many times independently, and it is believed to be present in well over 5% of vascular plant species (Winter and Smith, 1996; Silvera et al., 2010). These observations can be attributed to the fact that CAM most likely evolved on a biochemistry-first, anatomy-second trajectory (Edwards, 2019) in which the C_3_ + CAM phenotype is an evolutionarily accessible phenotype on the trajectory to strong CAM (Edwards and Donoghue, 2006; Bräutigam et al., 2017; Heyduk et al., 2018).

Our model demonstrates the water-saving potential of a partial CAM or CAM-like mode for plants grown under a broad range of conditions while maintaining a high net metabolic output; at 80% productivity, relative water saving is at least 25.4% across all conditions tested (up to 92.4%). It also shows that in terms of absolute water saving, the introduction of a classic CAM cycle is most beneficial for hot and dry conditions with high light and long days (scenario A), emphasizing the growth benefit of CAM plants in these climates. Much less intuitively, the model also revealed a significant water-saving potential for temperate climates and dense cropping environments in which lower light intensities prevail (scenarios B and C). Notably, under these conditions, the introduction of a CAM + isocitrate-citrate-Pro-2OG cycle would be most beneficial (contributing up to 10.6% of the total water saving) and therefore should be targeted at crops growing under such conditions. Despite the lower total water-saving potential in temperate climates, the high contribution of the isocitrate-citrate-Pro-2OG cycle to water saving could make a substantial contribution to water saving in environments where C_3_ crops are typically grown.

### Strategies to Engineering an Isocitrate-Citrate-Pro-2OG Cycle

In our previous work (Shameer et al., 2018), a number of engineering interventions beyond the core CAM cycle were identified, particularly concerning mitochondrial respiratory capacity. This work identified an additional isocitrate-citrate-Pro-2OG carbon-fixing cycle operating alongside the conventional CAM cycle. This additional cycle includes reactions in the mitochondria, cytosol, and vacuole and involves mitochondria-to-cytosol transport of Pro and (iso)citrate. The enzymatic reactions involved are the mitochondrial proline dehydrogenase (PDH), δ1-pyrroline-5-carboxylate dehydrogenase (P5CDH), glutamate dehydrogenase (GDH), as well as the TCA cycle enzymes ICDH and aconitase (both in the mitochondria and cytosol), glutamate 5-kinase, δ1-pyrroline-5-carboxylate synthetase (P5CS), and pyrroline-5-carboxylate reductase. With the exception of ICDH, these reactions already proceed in their favorable direction or operate close to equilibrium. Hence, a crucial aspect to engineering this flux in transgenic plants would be to ensure a high-enough 2OG level to drive ICDH backward. Key to this would be generating sufficient nighttime Pro accumulation and degradation to Glu during the day. GDH activity would probably then be sufficient to generate 2OG in high-enough quantities to overcome the thermodynamic barrier of the reverse ICDH reaction. In *Nicotiana tabacum* and potato (*Solanum tuberosum*), increased Pro production has been achieved by overexpression of P5CS (Kishor et al., 1995; Hmida-Sayaria et al., 2005). Moreover, as with any attempt to engineer CAM, transgene expression/enzyme activity would have to be diel-regulated, as reviewed elsewhere (Borland et al., 2014; DePaoli et al., 2014; Yang et al., 2015) and here, including PDH and P5CDH. Replication of the precise temporal patterns of this cycle predicted by our model may not be necessary, but recent developments in synthetic gene regulatory systems make this increasingly feasible (Belcher et al., 2020).

### Increasing Midday Depression in C_3_ Plants

Another prediction of the model was the benefit of a cessation of CO_2_ uptake (i.e., stomata closure) around midday in a leaf with C_3_ anatomy. From a metabolic perspective, a potential strategy would be altering the tonoplast malate transporter (aluminum‐activated malate transporter) in a way that malate is only released for decarboxylation at this time of the day. However, its regulation during day and night is still unknown. Therefore, generating a synthetic aluminum‐activated malate transporter that could be switched on in a precise temporal fashion could be a key engineering target.

### Overcoming Vacuolar Storage Constraints by Increasing Cell Size

Our study identified the vacuolar storage capacity as a limiting factor for introducing CAM into a C_3_ plant. Comparison of models with different vacuolar storage capacities revealed that shifting from C_3_ to CAM leaf anatomy (i.e., introducing a 3.1-fold increase in vacuolar storage capacity) increased water saving by 19.8% at 80% productivity. Given this observation, engineering leaf anatomical parameters of a C_3_ leaf toward CAM architecture will be key to increasing water saving. How could this be achieved? Several anatomical traits, including cell size (as a proxy for vacuole size), percentage intercellular airspace (IAS), and tissue thickness, have been discussed in this context (Edwards, 2019). However, altering the latter two parameters could potentially limit photosynthesis in C_3_ mode due to reduced CO_2_ diffusion through the mesophyll and a lower internal CO_2 _concentration (Evans and von Caemmerer, 1996) and therefore disadvantage flexible CAM. Indeed, facultative CAM plants when operating in the C_3_ mode can have an internal CO_2 _concentration as low as 110 μmol mol^−1^ (Maxwell et al., 1997). Barrera Zambrano et al. (2014) proposed that *Clusia* species might overcome this problem by having a high percentage IAS in the spongy mesophyll for efficient CO_2_ diffusion in the C_3_ mode and large palisade cells for carboxylic acid storage in the CAM mode. They suggested this as a potential engineering strategy for transferring inducible CAM into a C_3_ plant (Barrera Zambrano et al., 2014); however, engineering such tissue differentiation into a C_3_ crop would be a major challenge. Of particular relevance is a study that attempted to increase leaf cell size by overexpressing a grape berry (*Vitis vinifera*) transcription factor (VvCEB1_opt_) in Arabidopsis (*Arabidopsis thaliana*) and *Nicotiana sylvestris* (Lim et al., 2018)*.* This approach increased cell size—but not number—in both species, with a 1.8- to 2.3-fold increase in the palisade mesophyll and a 2.0- to 2.5-fold increase in the spongy mesophyll in Arabidopsis*.* Assuming that the larger cell size is mainly driven by increased vacuolar volume, the reported increase would suffice to enable partial CAM with high water-saving potential. Besides increased cell size, the authors also reported a significant decrease in cell wall thickness, another feature typically observed in CAM plants (Yang et al., 2015). A follow-up study in Arabidopsis reported significant increases in cell size, succulence, and decreased IAS and WUE (Lim et al., 2020). Thus, (tissue-specifically) overexpressing *VvCEB1*_*opt*_ in the context of engineering CAM could indeed be a promising strategy to engineer more drought-resistant crops.

### Further Challenges To Be Considered

Besides the challenge of engineering a CAM cycle into a C_3_ crop, other factors such as the trade-off between improved WUE and potential constraints on leaf productivity due to anatomical changes or the response of CAM to increasing CO_2_ levels need consideration (Winter, 2019). It is unclear not only how increased vacuolar size would affect the space available for enzymatic capacity in other intracellular compartments but also how it would affect mesophyll conductance. There is also the potential extra burden of increased structural costs (e.g., due to increased leaf thickness and altered water potential). Another challenge is that there may be other regulatory considerations that could affect the successful integration of CAM in C_3_ crops. For example, highly succulent CAM plants such as cacti avoid water stress in their photosynthetic tissues due to a regulatory response involving root shrinkage and metering of stored water in the succulent tissues (Nobel and Cui, 1992). The extent to which CAM can operate when tissues are water stressed to the levels routinely experienced by C_3_ leaves remains an open question. In conclusion, this study demonstrates the water-saving potential of introducing CAM-like metabolism into C_3_ plants under a wide range of environmental conditions and suggests environment-specific engineering targets for improved drought resistance.

## METHODS

### Developing a Time-Resolved Environment-Coupled Model of Leaf Metabolism

The model-building process was divided into two parts: developing a time-resolved model of leaf metabolism and modeling gas exchange through the stomata. The time-resolved diel model is an extension of our previously published diel modeling framework (Cheung et al., 2014; Shameer et al., 2018). Starting from the latest version of a charge- and proton-balanced generic core model of plant metabolism (PlantCoreMetabolism_v1_2_3.xml), we concatenated 24 copies of the model (each representing 1 h of the day) by allowing a range of metabolites to accumulate and to be transferred from one time point to the other via so-called linker reactions. Starch was allowed to accumulate freely in the plastid. The sugars glucose, sucrose, and fructose, the carboxylic acids malate, citrate, and isocitrate (see comment on citrate and isocitrate accumulation in Supplemental Methods, Section 1.2.1), the proteinogenic amino acids, and nitrate were allowed to accumulate in the vacuole. The last time interval of the optimization routine was coupled to the first interval to form a closed diel cycle. A comparison of of our previous and current model can be found in the Supplemental Table.

The overall vacuolar storage capacity was based on estimates for the vacuolar malate storage capacity and the vacuolar volume of an average C_3_ plant (Supplemental Methods, Section 1.2.4). The specificity of the metabolic network at each hour of the day was achieved by setting the light input according to the diel light curve and by constraining the export of sugars and amino acids to the phloem (phloem output) to a day:night ratio of 3:1 and nitrogen uptake to a day:night ratio of 3:2, according to previous estimates (Cheung et al., 2014). Maintenance cost was modeled in a light-dependent manner, where the daytime cost depends on the average daytime light intensity (Supplemental Methods, Section 1.2.3). The day:night ratio was assumed to be 3:1, and the ratio of ATP maintenance cost to NADPH maintenance cost was assumed to be 3:1 (Cheung et al., 2013). Rubisco was only activated during daylight hours. Since the applied normally distributed light curve reaches zero only asymptotically, calculated light intensities below a light-compensation point of 30 μmol m^−2^ s^−1^ (measured for *Kalanchoe daigremontiana*; Adams et al., 1987) were set to 0 μmol m^−2^ s^−1^ and the respective time interval was considered as dark.

Due to the lack of knowledge about the Rubisco carboxylation:oxygenation ratio at different steps on the Pareto frontier and for different time points during the day, we used a value of 3:1 based on flux measurements in Arabidopsis (*Arabidopsis thaliana*; Ma et al., 2014). The uptake rate for CO_2_ was limited to a value of 15 μmol m^−2^ s^−1^ based on values for different C_3_ and CAM species (Nobel, 2012). We assumed an average ratio of internal to atmospheric CO_2_ concentration of 0.7 (Tan et al., 2017; Kumarathunge et al., 2019; Busch, 2020; Cai et al., 2020; see Supplemental Methods, Section 1.2.5, and Supplemental Results, Section 2.5, for more information and a sensitivity analysis of the predicted water loss as dependent on the internal CO_2_ concentration). All other fluxes were unconstrained. To avoid any bias, we used phloem compositions of both C_3_ plants (tomato [*Solanum lycopersicum*] and Arabidopsis) and a CAM species (prickly pear [*Opuntia ficus-indica*]) and we found no qualitative differences in our analysis. The phloem composition and output values for the Arabidopsis and prickly pear data-constrained model are listed in the Supplemental Data Set.

Gas exchange through the stomata was described by a linearized diffusion model that predicts the water loss depending on the metabolic model’s demand for CO_2_ at particular T and RH values. The input values for T and RH for each time point were calculated by using a skewed sine-curve function (Supplemental Methods, Section 1.2.2), and this allowed us both to model the shape of actual T and RH curves and to systematically scan a multidimensional parameter space by adjusting the function parameters accordingly.

The model equations for optimizing phloem output and water saving were solved as a linear optimization problem (L_1_ norm). The subsequent minimization of the metabolic flux sum was solved as a quadratic optimization problem (L_2_ norm) to select, from a possible set of multiple solutions, the one with the least variation in fluxes between time points. To exemplify this, consider a three-time-step model with the flux sequence [1, 1, 1], [2, 0, 1], and [3, 0, 0]. When applying the L_1_ measure, all three cases will be weighted with 3, although in the second and third cases more enzyme needs to be synthesized and degraded and therefore would be costlier. The L_2_ distance yields values of 3, 5, and 9 and would therefore prefer the flux distribution in which fluxes are equally split between the three phases. The code for constructing and solving the full 24-phase model takes around 2 min to run on a standard desktop computer, and the Pareto scan takes ∼20 min. A derivation of the gas-water exchange relationship through the stomata, any further modeling assumptions, parameter derivations, and auxiliary calculations are detailed in the Supplemental Methods, Section 1.1. The effect of varying T and RH profiles and of different time intervals is summarised in Supplemental Figures 5 and 6.

### Data Availability

All modeling-relevant code is available at https://github.com/nadinetoepfer/Toepfer_et_al_CAM_2020.

### Supplemental Data

**Supplemental Figure 1.** Weather data on a typical summer day in 2019 in Gatersleben, Germany.
**Supplemental Figure 2.** Cumulative ATP turnover for the three models considered.
**Supplemental Figure 3.** Overview of the environmental conditions analyzed.
**Supplemental Figure 4.** Heat maps illustrating water saving for different combinations of light intensity and daylengths and different combinations of RH_min_ and T_max_.
**Supplemental Figure 5.** Effect of varying T and RH profiles on the occurrence of the four phases of the CAM cycle and the main flux routes.
**Supplemental Figure 6.** Effect of different time intervals on the overall model behavior.
**Supplemental Table.** Comparison of our previous and current large-scale metabolic model of CAM.

**Supplemental Methods.**

**Supplemental Results.**

**Supplemental Data Set.** Environmental parameters, leaf parameters for different C_3_ and CAM species, phloem sap compositions, and modeling results.

## DIVE Curated Terms

The following phenotypic, genotypic, and functional terms are of significance to the work described in this paper:

ICDH Gramene: AT1G54340
ICDH Araport: AT1G54340

ATP CHEBI: CHEBI:15422

starch CHEBI: CHEBI:28017

PEPC Gramene: B8XPZ2
PEPC Araport: B8XPZ2

water CHEBI: CHEBI:15377

## References

[bib1] Adams, W.W., Osmond, C.B., Sharkey, T.D. (1987). Responses of two CAM species to different irradiances during growth and susceptibility to photoinhibition by high light. Plant Physiol. 83: 213–218. 16665205 10.1104/pp.83.1.213 PMC1056327

[bib2] Allen, D.K., Ohlrogge, J.B., Shachar-Hill, Y. (2009). The role of light in soybean seed filling metabolism. Plant J. 58: 220–234. 19077167 10.1111/j.1365-313X.2008.03771.x

[bib3] Allen, D.K., Young, J.D. (2013). Carbon and nitrogen provisions alter the metabolic flux in developing soybean embryos. Plant Physiol. 161: 1458–1475. 23314943 10.1104/pp.112.203299 PMC3585609

[bib4] Bar-Even, A., Noor, E., Lewis, N.E., Milo, R. (2010). Design and analysis of synthetic carbon fixation pathways. Proc. Natl. Acad. Sci. USA 107: 8889–8894. 20410460 10.1073/pnas.0907176107 PMC2889323

[bib5] Bar-Even, A., Noor, E., Milo, R. (2012). A survey of carbon fixation pathways through a quantitative lens. J. Exp. Bot. 63: 2325–2342. 22200662 10.1093/jxb/err417

[bib6] Barrera Zambrano, V.A., Lawson, T., Olmos, E., Fernández-García, N., Borland, A.M. (2014). Leaf anatomical traits which accommodate the facultative engagement of Crassulacean acid metabolism in tropical trees of the genus *Clusia*. J. Exp. Bot. 65: 3513–3523. 24510939 10.1093/jxb/eru022

[bib7] Belcher, M.S., Vuu, K.M., Zhou, A., Mansoori, N., Agosto Ramos, A., Thompson, M.G., Scheller, H.V., Loqué, D., Shih, P.M. (2020). Design of orthogonal regulatory systems for modulating gene expression in plants. Nat. Chem. Biol. 16: 857–865. 32424304 10.1038/s41589-020-0547-4

[bib8] Borland, A.M., Griffiths, H., Broadmeadow, M.S.J., Fordham, M.C., Maxwell, C. (1993). Short-term changes in carbon-isotope discrimination in the C_3_-CAM intermediate *Clusia minor* L. growing in Trinidad. Oecologia 95: 444–453. 28314023 10.1007/BF00321001

[bib9] Borland, A.M., Griffiths, H., Hartwell, J., Smith, J.A.C. (2009). Exploiting the potential of plants with Crassulacean acid metabolism for bioenergy production on marginal lands. J. Exp. Bot. 60: 2879–2896. 19395392 10.1093/jxb/erp118

[bib10] Borland, A.M., Hartwell, J., Weston, D.J., Schlauch, K.A., Tschaplinski, T.J., Tuskan, G.A., Yang, X., Cushman, J.C. (2014). Engineering Crassulacean acid metabolism to improve water-use efficiency. Trends Plant Sci. 19: 327–338. 24559590 10.1016/j.tplants.2014.01.006 PMC4065858

[bib11] Bräutigam, A., Schlüter, U., Eisenhut, M., Gowik, U. (2017). On the evolutionary origin of CAM photosynthesis. Plant Physiol. 174: 473–477. 28416703 10.1104/pp.17.00195 PMC5462059

[bib12] Bulović, A., Fischer, S., Dinh, M., Golib, F., Liebermeister, W., Poirier, C., Tournier, L., Klipp, E., Fromion, V., Goelzer, A. (2019). Automated generation of bacterial resource allocation models. Metab. Eng. 55: 12–22. 31189086 10.1016/j.ymben.2019.06.001

[bib13] Busch, F.A. (2020). Photorespiration in the context of Rubisco biochemistry, CO_2_ diffusion and metabolism. Plant J. 101: 919–939. 31910295 10.1111/tpj.14674

[bib14] Cai, C., Li, G., Di, L., Ding, Y., Fu, L., Guo, X., Struik, P.C., Pan, G., Li, H., Chen, W., Luo, W., Yin, X. (2020). The acclimation of leaf photosynthesis of wheat and rice to seasonal temperature changes in T-FACE environments. Glob. Change Biol. 26: 539–556. 10.1111/gcb.14830 31505097

[bib15] Cheung, C.Y.M., Poolman, M.G., Fell, D.A., Ratcliffe, R.G., Sweetlove, L.J. (2014). A diel flux balance model captures interactions between light and dark metabolism during day-night cycles in C_3_ and Crassulacean acid metabolism leaves. Plant Physiol. 165: 917–929. 24596328 10.1104/pp.113.234468 PMC4044858

[bib16] Cheung, C.Y.M., Williams, T.C.R., Poolman, M.G., Fell, D.A., Ratcliffe, R.G., Sweetlove, L.J. (2013). A method for accounting for maintenance costs in flux balance analysis improves the prediction of plant cell metabolic phenotypes under stress conditions. Plant J. 75: 1050–1061. 23738527 10.1111/tpj.12252

[bib17] Cockburn, W. (1985). Tansley Review No 1. Variation in photosynthetic acid metabolism in vascular plants: CAM and related phenomena. New Phytol. 101: 3–24. 33873823 10.1111/j.1469-8137.1985.tb02815.x

[bib18] Cushman, J.C. (2001). Crassulacean acid metabolism: A plastic photosynthetic adaptation to arid environments. Plant Physiol. 127: 1439–1448. 11743087 PMC1540176

[bib19] Cushman, J.C., Bohnert, H.J. (1997). Molecular genetics of Crassulacean acid metabolism. Plant Physiol. 113: 667–676. 12223634 10.1104/pp.113.3.667 PMC158184

[bib20] de Mattos, E.A., Herzog, B., Lüttge, U. (1999). Chlorophyll fluorescence during CAM-phases in *Clusia minor* L. under drought stress. J. Exp. Bot. 50: 253–261.

[bib21] DePaoli, H.C., Borland, A.M., Tuskan, G.A., Cushman, J.C., Yang, X. (2014). Synthetic biology as it relates to CAM photosynthesis: Challenges and opportunities. J. Exp. Bot. 65: 3381–3393. 24567493 10.1093/jxb/eru038

[bib22] Dodd, A.N., Borland, A.M., Haslam, R.P., Griffiths, H., Maxwell, K. (2002). Crassulacean acid metabolism: Plastic, fantastic. J. Exp. Bot. 53: 569–580. 11886877 10.1093/jexbot/53.369.569

[bib23] Edwards, E.J. (2019). Evolutionary trajectories, accessibility and other metaphors: The case of C_4_ and CAM photosynthesis. New Phytol. 223: 1742–1755. 30993711 10.1111/nph.15851

[bib24] Edwards, E.J., Donoghue, M.J. (2006). Pereskia and the origin of the cactus life-form. Am. Nat. 167: 777–793. 16649155 10.1086/504605

[bib25] Evans, J.R., Von Caemmerer, S. (1996). Carbon dioxide diffusion inside leaves. Plant Physiol. 110: 339–346. 12226185 10.1104/pp.110.2.339 PMC157726

[bib26] Farnsworth, K.D., Niklas, K.J. (1995). Theories of optimization, form and function in branching architecture in plants. Funct. Ecol. 9: 355.

[bib27] Garcia, T.M., Heyduk, K., Kuzmick, E., Mayer, J.A. (2014). Crassulacean acid metabolism biology. New Phytol. 204: 738–740. 25367608 10.1111/nph.13127

[bib28] Gawronska, K., Niewiadomska, E. (2015). Participation of citric acid and isocitric acid in the diurnal cycle of carboxylation and decarboxylation in the common ice plant. Acta Physiol. Plant. 37: 61.

[bib29] Goelzer, A., Muntel, J., Chubukov, V., Jules, M., Prestel, E., Nölker, R., Mariadassou, M., Aymerich, S., Hecker, M., Noirot, P., Becher, D., Fromion, V. (2015). Quantitative prediction of genome-wide resource allocation in bacteria. Metab. Eng. 32: 232–243. 26498510 10.1016/j.ymben.2015.10.003

[bib30] Gornall, J., Betts, R., Burke, E., Clark, R., Camp, J., Willett, K., Wiltshire, A. (2010). Implications of climate change for agricultural productivity in the early twenty-first century. Philos. Trans. R. Soc. Lond. B Biol. Sci. 365: 2973–2989. 20713397 10.1098/rstb.2010.0158 PMC2935125

[bib31] Heyduk, K., Ray, J.N., Ayyampalayam, S., Leebens-Mack, J. (2018). Shifts in gene expression profiles are associated with weak and strong Crassulacean acid metabolism. Am. J. Bot. 105: 587–601. 29746718 10.1002/ajb2.1017

[bib32] Hmida-Sayaria, A., Gargouri-Bouzida, R., Bidania, A., Jaoua, L., Savouré, A., Jaoua, S. (2005). Overexpression of Δ1-pyrroline-5-carboxylate synthetase increases proline production and confers salt tolerance in transgenic potato plants. Plant Sci. 169: 746–752.

[bib33] Holzhütter, H.-G. (2004). The principle of flux minimization and its application to estimate stationary fluxes in metabolic networks. Eur. J. Biochem. 271: 2905–2922. 15233787 10.1111/j.1432-1033.2004.04213.x

[bib34] Igamberdiev, A.U., Eprintsev, A.T. (2016). Organic acids: The pools of fixed carbon involved in redox regulation and energy balance in higher plants. Front. Plant Sci. 7: 1042. 27471516 10.3389/fpls.2016.01042 PMC4945632

[bib35] Kennedy, M.C. (2010). Functional-structural models optimize the placement of foliage units for multiple whole-canopy functions. Ecol. Res. 25: 723–732.

[bib36] Kishor, P., Hong, Z., Miao, G.H., Hu, C., Verma, D. (1995). Overexpression of [delta]-pyrroline-5-carboxylate synthetase increases proline production and confers osmotolerance in transgenic plants. Plant Physiol. 108: 1387–1394. 12228549 10.1104/pp.108.4.1387 PMC157516

[bib37] Kumarathunge, D.P., Medlyn, B.E., Drake, J.E., Rogers, A., Tjoelker, M.G. (2019). No evidence for triose phosphate limitation of light-saturated leaf photosynthesis under current atmospheric CO_2_ concentration. Plant Cell Environ. 42: 3241–3252. 31378950 10.1111/pce.13639

[bib38] Lantier, L., Williams, A.S., Williams, I.M., Yang, K.K., Bracy, D.P., Goelzer, M., James, F.D., Gius, D., Wasserman, D.H. (2015). SIRT3 is crucial for maintaining skeletal muscle insulin action and protects against severe insulin resistance in high-fat-fed mice. Diabetes 64: 3081–3092. 25948682 10.2337/db14-1810 PMC4542443

[bib39] Laundy, R.S., Steuer, R.E. (1988). Multiple criteria optimisation: Theory, computation and application. J. Oper. Res. Soc. 39: 879.

[bib40] Lewis, N.E., (2010). Omic data from evolved *E. coli* are consistent with computed optimal growth from genome-scale models. Mol. Syst. Biol. 6: 390. 20664636 10.1038/msb.2010.47 PMC2925526

[bib41] Lim, S.D., Mayer, J.A., Yim, W.C., Cushman, J.C. (2020). Plant tissue succulence engineering improves water-use efficiency, water-deficit stress attenuation and salinity tolerance in Arabidopsis. Plant J. 103: 1049–1072. 32338788 10.1111/tpj.14783

[bib42] Lim, S.D., Yim, W.C., Liu, D., Hu, R., Yang, X., Cushman, J.C. (2018). A *Vitis vinifera* basic helix-loop-helix transcription factor enhances plant cell size, vegetative biomass and reproductive yield. Plant Biotechnol. J. 16: 1595–1615. 29520945 10.1111/pbi.12898 PMC6096725

[bib43] Lopez, H., West, R., Dong, S., Goni, G., Kirtman, B., Lee, S.-K., Atlas, R. (2018). Early emergence of anthropogenically forced heat waves in the western United States and Great Lakes. Nat. Clim. Chang. 8: 414–420.

[bib44] Lüttge, U. (1988). Day-night changes of citric-acid levels in Crassulacean acid metabolism: Phenomenon and ecophysiological significance. Plant Cell Environ. 11: 445–451.

[bib45] Lüttge, U. (1990). Nocturnal citrate accumulation and its response to environmental stress in the CAM plant *Kalanchoe pinnata* (Lam.) Pers. Plant Cell Environ. 13: 977–982.

[bib46] Lüttge, U. (2004). Ecophysiology of Crassulacean acid metabolism (CAM). Ann. Bot. 93: 629–652. 15150072 10.1093/aob/mch087 PMC4242292

[bib47] Ma, F., Jazmin, L.J., Young, J.D., Allen, D.K. (2014). Isotopically nonstationary ^13^C flux analysis of changes in *Arabidopsis thaliana* leaf metabolism due to high light acclimation. Proc. Natl. Acad. Sci. USA 111: 16967–16972. 25368168 10.1073/pnas.1319485111 PMC4250135

[bib48] Maclennan, D.H., Beevers, H., Harley, J.L. (1963). ‘Compartmentation’ of acids in plant tissues. Biochem. J. 89: 316–327. 16749046 10.1042/bj0890316 PMC1202364

[bib49] Maxwell, K., von Caemmerer, S., Evans, J.R. (1997). Is a low internal conductance to CO_2_ diffusion a consequence of succulence in plants with Crassulacean acid metabolism? Funct. Plant Biol. 24: 777–786.

[bib50] Nobel, P.S. (2012). Physicochemical and Environmental Plant Physiology.. (Cambridge, Massachusetts: Academic Press).

[bib51] Nobel, P.S., Cui, M. (1992). Shrinkage of attached roots of *Opuntia ficus-indica* in response to lowered water potentials: Predicted consequences for water uptake or loss to soil. Ann. Bot. (Lond.) 70: 485–491.

[bib52] Olesen, J.E., Bindi, M. (2002). Consequences of climate change for European agricultural productivity, land use and policy. Eur. J. Agron. 16: 239–262.

[bib53] Osmond, C.B. (1978). Crassulacean acid metabolism: A curiosity in context. Annu. Rev. Plant Physiol. 29: 379–414.

[bib54] Rasmijn, L.M., van der Schrier, G., Bintanja, R., Barkmeijer, J., Sterl, A., Hazeleger, W. (2018). Future equivalent of 2010 Russian heatwave intensified by weakening soil moisture constraints. Nat. Clim. Chang. 8: 381–385.

[bib55] Reimers, A.-M., Knoop, H., Bockmayr, A., Steuer, R. (2017). Cellular trade-offs and optimal resource allocation during cyanobacterial diurnal growth. Proc. Natl. Acad. Sci. USA 114: E6457–E6465. 28720699 10.1073/pnas.1617508114 PMC5547584

[bib56] Roberts, A., Borland, A.M., Griffiths, H. (1997). Discrimination processes and shifts in carboxylation during the phases of Crassulacean acid metabolism. Plant Physiol. 113: 1283–1292. 12223674 10.1104/pp.113.4.1283 PMC158251

[bib57] Rügen, M., Bockmayr, A., Steuer, R. (2015). Elucidating temporal resource allocation and diurnal dynamics in phototrophic metabolism using conditional FBA. Sci. Rep. 5: 15247. 26496972 10.1038/srep15247 PMC4620596

[bib58] Sanada, Y., Ueda, H., Kuribayashi, K., Andoh, T., Hayashi, F., Tamai, N., Wada, K. (1995). Novel light-dark change of proline levels in halophyte (*Mesembryanthemum crystallinum* L.) and glycophytes (*Hordeum vulgare* L. and *Triticum aestivum* L.) leaves and roots under salt stress. Plant Cell Physiol. 36: 965–970.

[bib59] Schwender, J., Shachar-Hill, Y., Ohlrogge, J.B. (2006). Mitochondrial metabolism in developing embryos of *Brassica napus*. J. Biol. Chem. 281: 34040–34047. 16971389 10.1074/jbc.M606266200

[bib60] Shameer, S., Baghalian, K., Cheung, C.Y.M., Ratcliffe, R.G., Sweetlove, L.J. (2018). Computational analysis of the productivity potential of CAM. Nat. Plants 4: 165–171. 29483685 10.1038/s41477-018-0112-2

[bib61] Silvera, K., Neubig, K.M., Whitten, W.M., Williams, N.H., Winter, K., Cushman, J.C. (2010). Evolution along the Crassulacean acid metabolism continuum. Funct. Plant Biol. 37: 995.

[bib62] Smil, V. (2003). The Earth’s Biosphere: Evolution, Dynamics, and Change.. (Cambridge, MA: MIT Press).

[bib63] Tan, Z.H., (2017). On the ratio of intercellular to ambient CO2 (*c*i/*c*a) derived from ecosystem flux. Int. J. Biometeorol. 61: 2059–2071. 28707041 10.1007/s00484-017-1403-4

[bib64] Wai, C.M., Weise, S.E., Ozersky, P., Mockler, T.C., Michael, T.P., VanBuren, R. (2019). Time of day and network reprogramming during drought induced CAM photosynthesis in *Sedum album*. PLoS Genet. 15: e1008209. 31199791 10.1371/journal.pgen.1008209 PMC6594660

[bib65] Winter, K. (2019). Ecophysiology of constitutive and facultative CAM photosynthesis. J. Exp. Bot. 70: 6495–6508. 30810162 10.1093/jxb/erz002

[bib66] Winter, K., Lüttge, U., Winter, E., Troughton, J.H. (1978). Seasonal shift from C_3_ photosynthesis to Crassulacean acid metabolism in *Mesembryanthemum crystallinum* growing in its natural environment. Oecologia 34: 225–237. 28309551 10.1007/BF00345168

[bib67] Winter, K., Smith, J.A.C. (1996). Crassulacean acid metabolism: Current status and perspectives. In Crassulacean Acid Metabolism, K. Winter, and J.A.C. Smith, eds (Berlin: Springer), pp. 389–426.

[bib68] Yang, X., (2015). A roadmap for research on Crassulacean acid metabolism (CAM) to enhance sustainable food and bioenergy production in a hotter, drier world. New Phytol. 207: 491–504. 26153373 10.1111/nph.13393

